# Tendon transfers in BPBP

**DOI:** 10.1186/1753-6561-9-S3-A18

**Published:** 2015-05-19

**Authors:** Hari Venkatramani

**Affiliations:** 1 Ganga Hospital, Coimbatore, 641043, India

## Tendon transfers in birth brachial plexus palsy

Many patients with birth brachial plexus palsy (BBPP) have good spontaneous recovery. This is because the injury is a low velocity injury compared to the adult palsy and hence the injury is mostly a stretch injury which has high potential to recover. Even if there is rupture of the roots the distance to bridge and to travel to the destination is closer resulting in good spontaneous recovery. For the patients with insufficient recovery, nerve surgeries are indicated. Tendon transfers procedures are recommended when the child is not a candidate for nerve surgery operation. Additionally, tendon transfers are commonly required to improve the existing function after insufficient spontaneous recovery or after nerve repair surgery. Tendon transfers are commonly done to improve the shoulder function and less frequently to restore elbow flexion or extension.

## Improvement of shoulder function

Tendon transfers are commonly done to improve shoulder abduction and external rotation. Most children with BBPP do have good spontaneous recovery of the shoulder abductors but they are not able to act to their full potential because of the tight and abnormally co-contracting adductors. Release of adductors will improve the shoulder abduction in these patients. If the external rotation is to be restored latissimus dorsi can be transferred to teres minor. In patients with absent passive external rotation we prefer to do a bony procedure to improve shoulder external rotation at a second stage as we have found that anterior shoulder release when combined with external rotation augmentation procedure results in disabling loss of internal rotation.

Patients with totally paralyzed or very weak shoulder abductors are less commonly encountered. They require augmentation of the abduction power with a trapezius to deltoid transfer.

## Restoration of elbow flexion

The options include- latissimus dorsi transfer, pectoralis transfer, Steindler's procedure, triceps to biceps transfer and free functioning gracilis muscle transfer. Triceps to biceps transfer works well in patients who have significant co-contraction between these muscles and the result is almost instantaneous but the elbow extension is lost. Latissimus dorsi and pectoralis may be weak in patients with birth brachial plexus palsy and require detailed assessment of their power. They both work well although results with the latissimus dorsi are more superior than those utilising the pectoralis. Pectoralis major transfer is also not recommended in female patients for the fear of breast asymmetry.

Steindler's procedure works well in patients with strong wrist extensors and wrist and finger flexors but results in loss of supination movement and a 20 degree of fixed flexion deformity at the elbow. Free functioning gracilis is a good option and adds to the existing power of the limb instead of sharing the available muscle power. If there is the slightest doubt concerning the existing power of the donor muscles in the ipsilateral limb we should resort to free functioning muscle transfers.

## Restoration of elbow extension

This is a less common indication for doing tendon transfers. Absence of elbow extension disables the patient by reducing the reach for the objects and it becomes even more disabling if the shoulder is weak and unable to compensate for the lack of elbow extension. We have found that restoration of elbow extension in patients with reasonable hand function greatly improves the working space of the hand and overall hand function. We have utilised two techniques: in patients with good deltoid function, the posterior half of the deltoid can be transferred to the triceps and in patients with weak deltoid, the lower trapezius can be transferred to triceps to restore elbow extension.

